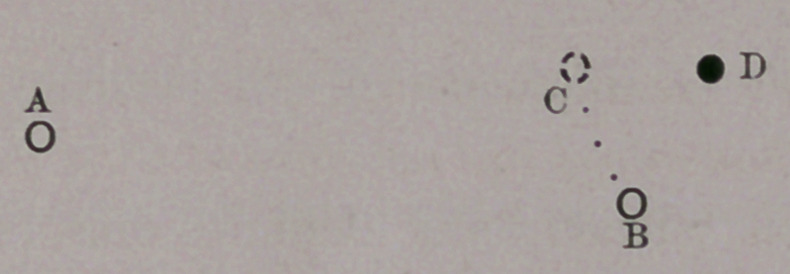# Curious Case of Double and Eccentric Vision—Cured by Two Doses of Sulphur CM

**Published:** 1893-07

**Authors:** Thomas Skinner

**Affiliations:** London


### CURIOUS CASE OF DOUBLE AND ECCENTRIC VISION—CURED BY TWO DOSES OF SULPHUR CM (F. C.).

THOMAS SKINNER, M. D., LONDON.

Mr. E. H., a Swiss merchant, living in London, consulted me on the 18th of February, 1893. The gentleman is a famous amateur billiard player, and spends most of his evenings at home playing billiards with his brother and friends.

He complains much of his *left* eye, having for about three years seen double with it, on and off. At times—say when playing billiards—he feels as if there were something in the left eye, and when looking at the three balls on the table, if standing erect, he sees them all in their exact relative positions, but as soon as he takes his cue and stoops to play a cannon or pocket a ball, the ball he is going to cannon off, moves, or to his vision seems to move, in line with the other ball he means to cannon off. Of course, when this occurs, and it has occurred alas, too often of late, billiards, so far as my patient is concerned, is out of the question. On again standing erect he sees the relative position of the balls as they should be. In his own words : "The change of position of the ball takes place only when I stoop to use my cue in playing the cannon."

This rude diagram will show at once what occurs. A represents E. H.'s ball; B his opponent's, and D the red ball. As soon as he aims with his cue at B, and before he has touched A, B seems to him to have moved to C—which is one of those things "which no fellah can understand."

The game is up for that night. E. H. has consulted several oculists, but all that he ever got out of them was a learned dissertation as to how such an aberration of vision occurred. All agreed that it was a rare occurrence! I may just as well add that the color of the ball, red or white, had nothing to do with the morbid phenomena of vision.

I candidly confess that at first I felt myself quite as much at sea as the learned and scientific oculists. I could make nothing of the mere obliquity of vision. I could find it nowhere—not even in Berridge's *Eye Repertory*—so I asked the patient if he had or had had anything else the matter with him?

*Past History.*—Sometime ago he had a swelling of the right back of the neck, night and morning, accompanied with a neuralgic pain in left temple, which lately has become greatly exaggerated, and is always bad when this eccentric vision is troublesome. *He had also a discharge of pus from his left ear, greenish and offensive.* Once last autumn he had a mountain climb in Switzerland, when he was up to the hips in snow for six hours, and he does not think that that did him much good.

*Lastly.—The discharge from the left ear was suppressed by local measures, and he has sinking at the epigastrium daily at eleven A. M.*

*February* 18*th*, 1893.—*Sulphur*$^{cm}$ (F. C.), one dose, stationed dry on his tongue and another at bed-time.

*March* 15*th*, 1893.—Double vision gone. No change in position of billiard ball. None whatever since February 18th.

Sinking at eleven A. M. gone. Much belching then instead. S. L. *ad libitum.*

*June 5th,* 1893.—Since early in April my patient is quite well, and he has not felt so fit and well for years before, and this is the result of constitutional treatment *versus* local measures. Suppressed discharge. *Sulphur to the rescue.*

## Figures and Tables

**Figure f1:**